# Association Between Maternal Weight Perception Before and During Pregnancy and Postpartum Depression Status in Southern China

**DOI:** 10.3390/nu16213696

**Published:** 2024-10-30

**Authors:** Qin Zhang, Menglu Qiu, Laidi Guo, Yuanyuan Li, Zhencheng Xie, Wanyi Yang, Lishan Ouyang, Jia Yin, Yun Zhou, Minghan Fu, Ye Ding, Zhixu Wang

**Affiliations:** 1 Department of Maternal, Child and Adolescent Health, School of Public Health, Nanjing Medical University, Nanjing 211166, China; zqin@stu.njmu.edu.cn (Q.Z.); menglu-qiu@stu.njmu.edu.cn (M.Q.); guolaidi@stu.njmu.edu.cn (L.G.); lyy272829@stu.njmu.edu.cn (Y.L.); zhenchengxie@njmu.edu.cn (Z.X.); yangwanyi@stu.njmu.edu.cn (W.Y.); ouyanglishan@stu.njmu.edu.cn (L.O.); yinjia0819@stu.njmu.edu.cn (J.Y.); zygw@stu.njmu.edu.cn (Y.Z.); fuminghan@stu.njmu.edu.cn (M.F.); 2 The Institute of Nutrition and Food Science, Nanjing Medical University, Nanjing 211166, China

**Keywords:** pre-pregnancy weight, gestational weight gain, weight perception, postpartum depression

## Abstract

Objective: Postpartum depression (PPD) is a common complication after childbirth. Weight misperception can lead to self-esteem issues and mental health problems, especially in women and adolescents. The aim of this study was to investigate the association between weight perception before and during pregnancy and the status of PPD in Southern China. Methods: From October 2021 to November 2023, a multi-stage sampling method was used to recruit 2169 eligible mothers aged 18–49 who had delivered live-born singleton infants within 3 to 180 days postpartum. Anthropometric measurements and face-to-face questionnaire surveys were conducted to collect data. The Kappa test was used to assess the agreement between actual and perceived weight. The generalized linear model incorporating multiplicative interaction analysis was applied to explore the associations among variables. Results: The prevalence of PPD status was 18.0%. Among women, 35.2% perceived their pre-pregnancy weight (PPW) as abnormal, while 33.1% perceived their gestational weight gain (GWG) as inappropriate. There was poor agreement between maternal actual and perceived PPW/GWG (Kappa = 0.366, *p* < 0.001; Kappa = 0.188, *p* < 0.001), with 27.8% of women misperceiving their PPW and 52.1% misperceiving their GWG. The results of the general linear model indicated that women who perceived their PPW as underweight (*β* = 0.70, *p* = 0.016) or overweight/obese (*β* = 0.86, *p* < 0.001), as well as those who perceived their GWG as excessive (*β* = 0.47, *p* = 0.028) were more likely to exhibit PPD status. The interaction analysis results showed that those who perceived their PPW as underweight and their GWG as insufficient (*β* = 1.75, *p* = 0.020), as well as those who perceived their PPW as overweight/obese and their GWG as excessive (*β* = 0.90, *p* = 0.001) had a positive interactive effect on the occurrence of PPD status, while underestimating PPW and GWG may be a protective factor against PPD status (*β* = −1.03, *p* = 0.037). Conclusion: These findings support that maternal weight perception plays a role in the development of PPD status. Further improvement is needed in personalized health education for weight management, both prior to and throughout the pregnancy period. This can help women reduce weight anxiety, better understand their body image, and potentially lower the risk of developing PPD.

## 1. Introduction

Postpartum depression (PPD), the most common complication after childbirth, is emerging as a significant global health concern. It encompasses symptoms such as depressive mood, anhedonia, heightened anxiety, sleep disorders, reduced appetite, concentration difficulties, and a lack of interest in daily activities. In severe cases, PPD may also involve suicidal thoughts [1]. On average, the global incidence of PPD is 17.22% [2]. In China, a meta-analysis spanning 2014 to 2022 showed that the detection rate of PPD was 16% [3]. The immediate effect of PPD is the deterioration of women’s physical and mental well-being [4]. Additionally, it indirectly affects the growth and development as well as emotional maturity of infants by affecting breastfeeding practices and mother–infant interaction [5,6]. In the long term, mothers with PPD are more susceptible to the onset of new psychiatric disorders, experiencing increased levels of mood symptoms and facing a decline in their quality of life and functional capabilities [7]. Children who are continuously exposed to maternal depressive symptoms are at an increased risk of cognitive delays, behavioral issues, and higher rates of adolescent anxiety or depression [8,9].

Weight perception denotes the assessment undertaken by a person regarding their own weight status [10]. Weight misperception refers to the discordance that exists between perceived and actual weight levels [11]. Current research indicates that the general public often fails to accurately perceive their weight, with a common tendency to overestimate or underestimate it [12,13]. Despite the absence of direct evidence linking weight perception to PPD, studies among adolescents have shown that inaccurate estimation of body weight can lead to increased risks of self-esteem issues and may trigger mental health problems, such as depression and anxiety [14]. Additionally, influenced by social and cultural factors, a higher proportion of women are concerned about their body image compared to men, and they also exhibit greater sensitivity to depression and stress [15]. Body image is a crucial component of self-representation, referring to an individual’s perception and evaluation of their physical appearance, with weight perception being its core [16]. Various researchers have demonstrated that women who express dissatisfaction with their body image tend to exhibit a strong positive correlation with the symptoms of depression they report [17,18].

Pregnancy is a critical period characterized by significant and rapid changes in the size and shape of a woman’s body, spanning approximately ten months. With maternal and child health being the paramount concern, weight monitoring has become a central focus before conception, throughout pregnancy, and postpartum. Pregnant individuals are often subjected to a barrage of weight-related information and expectations and require regular weigh-ins to assess the adequacy of their weight gain. This monitoring, in turn, will exacerbate the risk of body dissatisfaction and the emergence of associated maladaptive cognitions [19]. Studies have shown that the majority of women maintain a negative view of their body image after giving birth [20]. Roomruangwong C’s research has pointed out that pregnant participants who express dissatisfaction with their body image, which is associated with increased anxiety and depression, may serve as an indication of an underlying mood disorder [21]. Therefore, it is necessary to study the association between pregnant women’s perceptions of their weight status before and during pregnancy and the psychological issues they may encounter. However, existing research on the relationship between weight perception and psychological well-being among pregnant women is limited.

At present, studies on pregnant women have only considered the factors of maternal weight perception (feeling thin, normal, or overweight/obese), but there is limited research on the potential harm caused by maternal weight misperception [22]. It is universally acknowledged that proper weight management during pregnancy is important: excessive or insufficient gestational weight gain (GWG) can have a negative impact on the mother’s physical and mental health, as well as the growth and development of the fetus [23,24]. Therefore, understanding the relationship between body weight perception agreement, including pre-pregnancy weight (PPW) and GWG, and PPD status is important for developing effective interventions to prevent and manage PPD. Furthermore, the risk factors for PPD are intricate and multifaceted. Sociodemographic factors, obstetrical factors, and lifestyle were the most commonly identified environmental risk factors and need to be taken into account [25,26,27]. Accordingly, this study conducted an extensive cross-sectional survey among postpartum women of Southern China, aiming to explore the potential association between body weight misperception (including PPW and GWG) and PPD status after controlling for confounding factors.

## 2. Methods

### 2.1. Study Participants and Design

A multi-stage sampling method was used to recruit the study participants. To begin with, Guangdong Province and Guangxi Zhuang Autonomous Region were selected as representatives in Southern China by random sampling. Subsequently, each province (autonomous region) was divided into urban and rural areas. Finally, two maternal and child health institutions from urban or rural areas of each province (autonomous region) were randomly selected as survey sites for data collection. Given that Maternity and Child Health Care of Guangxi Zhuang Autonomous Region has two branches, each located in a different district, the two branches were treated as separate maternal and child care institutions to recruit participants in this study. In the end, a total of 7 maternal and child health institutions were selected.

From October 2021 to November 2023, mothers aged 18 to 49 years within 3~180 days after childbirth were recruited if they had delivered live-born singleton infants and had resided in the region for more than 12 months. Mothers whose weight data were unavailable at any stage (pre-pregnancy, first trimester, or prior to delivery), as well as those who experienced challenges with speech communication or had mental disorders, were excluded. Ethical approval for this study was provided by the Medical Ethics Committee of Tongji Medical College, Huazhong University of Science and Technology (No. 2021-S092), and the study was also registered with the Chinese Clinical Trials Registry (ChiCTR2100051019). Written informed consent was obtained from all participants.

Our study’s sample size was determined using the following formula: $n=\frac{Z_{\propto }^{2}(1-P)}{\varepsilon^{2}P}$, where Z denotes the critical value for a 95% confidence level (*Z_α_* = 1.96), *ε* represents the maximum relative error (10%), and *P* is the prevalence of PPD status among Chinese women, estimated at 16.0% based on a recent meta-analysis of 18 studies from 2014 to 2022 [3]. This calculation indicated a minimum requirement of 2017 participants. Ultimately, our study enrolled a total of 2169 women, meeting the target sample size.

### 2.2. Processes of Data Acquisition

#### 2.2.1. Maternal Actual Weight

The data on sociodemographic characteristics and clinical factors were derived from basic information questionnaires and electronic medical records. Among them, PPW data were self-reported by the participants, while the weight data in the first trimester or before delivery, along with the height data, were measured by medical staff using standard measuring instruments.

Referring to the PPW prediction model developed by Thomas [28], the maternal self-reported PPW was calibrated. When the difference between the self-reported data and the calculated data was greater than ±2 kg, the PPW calculated by the Thomas formula was used instead of the self-reported value. The Thomas formula is as follows:

Y = 6.10 + 0.99 x_1_ − 0.01 x_2_ − 0.02 x_3_ − 0.04 x_4_ − 0.09 x_5_

where Y = PPW (kg)

- x_1_ = first trimester measured weight (kg);
- x_2_ = gestational age (days) at first weight measurement;
- x_3_ = height (cm);
- x_4_ = maternal age at conception (years);
- x_5_ = parity.

The pre-pregnancy BMI was calculated based on maternal PPW and height. According to the Chinese Health Industry Standard “Adult Weight Determination” (WS/T428-2013) [29], the pre-pregnancy BMI was divided into underweight (BMI < 18.5 kg/m^2^), normal weight (18.5 kg/m^2^ ≤ BMI < 24.0 kg/m^2^), overweight (24.0 kg/m^2^ ≤ BMI < 28.0 kg/m^2^), and obese (BMI ≥ 28.0 kg/m^2^). According to the Chinese Health Industry Standard “Recommendation for Weight Gain during Pregnancy Period” (WS/T801-2022) [30], optimal weight gain ranges for single-pregnancy women with different pre-pregnancy BMI conditions were established. The optimal ranges for weight gain during pregnancy with pre-pregnancy underweight, normal weight, overweight, and obesity are 11~16 kg, 8~14 kg, 7~11 kg, and 5~9 kg, respectively.

#### 2.2.2. Maternal Weight Perception

The perception of PPW (1 item) was assessed with the question, “How do you perceive your PPW?” Answers were categorized into ‘underweight’, ‘normal weight’, and ‘overweight/obesity’. Similarly, the perception of GWG (1 item) was assessed with the question, “How do you perceive your weight gain during pregnancy?” Answers were categorized into ‘insufficient’, ‘appropriate’, and ‘excessive’.

The agreements between actual and perceived PPW, as well as between actual and perceived GWG, were classified into three categories: underestimation, consistency, and overestimation. Taking PPW as an example, underestimation refers to the perceived weight being lower than the actual weight. Consistency occurs when the perceived weight is consistent with the actual weight. Overestimation, on the other hand, refers to the perceived weight being higher than the actual weight.

#### 2.2.3. PPD Status

The Edinburgh Postnatal Depression Scale (EPDS) was used to assess whether postpartum women had experienced PPD status within the past week. The Chinese version of the EPDS used in this study has favorable reliability and validity, with a reported internal consistency Cronbach’s α coefficient of 0.790 and a Guttman’s split-half coefficient of 0.760 [31]. The EPDS comprises 10 items, each of which is graded across four levels, with assigned scores varying from 0 to 3. These 10 individual scores are cumulatively calculated, resulting in the EPDS score that spans a range of 0 to 30. An EPDS score of >10 signifies PPD status; higher scores indicate a more severe depressive condition.

#### 2.2.4. Covariates

Covariates included maternal postpartum age, ethnicity, region of residence, household income, education level, employment, parity, mode of delivery, and gestational age at birth. Notably, only 2 participants reported smoking, while 9 participants reported drinking in this study. Therefore, only passive smoking status was listed as a covariate.

In addition, physical activity level was also considered a covariate that needs to be adjusted in this study. It was assessed through a separate self-administered tool, namely the International Physical Activity Questionnaire-Short (IPAQ-S). The Chinese version of the IPAQ-S used in this study has been tested to have moderate reliability and validity. The intra-class correlation coefficients of energy expenditure across various physical activities exhibited a range of 0.51 to 0.80, and the Spearman correlation coefficient of energy expenditure recorded by IPAQ-S and physical activity log fluctuated within a range of 0.44 to 0.58 [32]. The questionnaire consisted of 7 items and used indicators in metabolic equivalents (METs) to assign physical activity levels. Based on IPAQ guidelines, physical activity levels were categorized into three grades: low, moderate, and high.

### 2.3. Statistical Analysis

All analyses were conducted in SPSS 26.0 software (IBM Corp., Armonk, NY, USA). Categorical variables were presented in the form of frequency (*n*) and percentage (%) and underwent analysis through the chi-squared test (χ^2^) to examine any potential differences. Continuous variables with non-normal distribution were presented as median (*P*_25_, *P*_75_) and were subjected to the Mann–Whitney U rank-sum test for comparison. The Kappa test was used to assess the agreements between actual and perceived PPW, as well as between actual and perceived GWG. The Kappa coefficient was calculated and interpreted as follows: ≤0 = poor; 0.01 to 0.20 = slight; 0.21 to 0.40 = fair; 0.41 to 0.60 = moderate; 0.61 to 0.80 = substantial; and 0.81 to 1.00 = almost perfect. The generalized linear model was used to separately test the associations between actual PPW, perceived PPW, and the results of their agreement with PPD status among postpartum women, as well as between actual GWG, perceived GWG, and the results of their agreement with PPD status in the same population. The multiplicative interaction between PPW perception and GWG perception on the risk of PPD status, as well as the multiplicative interaction between the actual and perceived agreement of PPW and that of GWG on the risk of PPD status, were further explored separately. Analyses were adjusted for maternal postpartum age, region of residence, household income, education level, physical activity level, and parity. All results were deemed statistically significant with a *p*-value less than 0.05.

## 3. Results

### 3.1. Sociodemographic Characteristics

The prevalence of PPD status was 18.0% among postpartum women in Southern China. As shown in Table 1, a comparative analysis revealed that, compared with the non-PPD group, the PPD group had a significantly higher proportion of individuals under 35 years old (88.7% vs. 82.4%), urban residents (62.4% vs. 45.4%), those with per capita income between RMB 5000 and RMB 10,000 (38.6% vs. 25.0%), individuals with bachelor’s degrees or above (27.4% vs. 22.8%), primiparous women (57.8% vs. 47.5%), and those engaging in lower levels of physical activity (61.6% vs. 43.1%). All these differences were statistically significant (*p* < 0.05).

### 3.2. Agreements Between Maternal Actual and Perceived PPW

**Figure 1:**
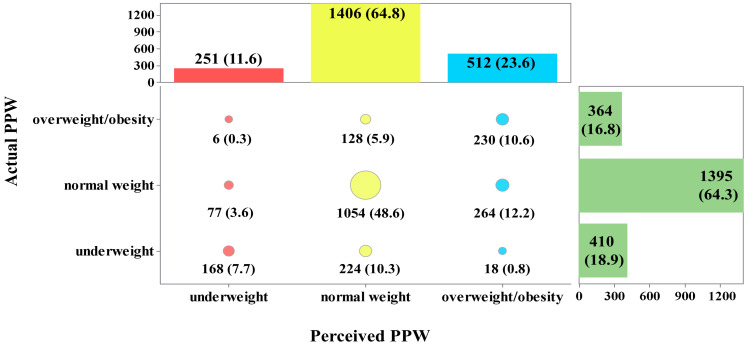
Proportions of exact agreement between maternal actual and perceived PPW. PPW, pre-pregnancy weight. Red circles: the proportion of participants who perceived pre-pregnancy underweight. Yellow circles: the proportion of participants who perceived pre-pregnancy normal weight. Blue circles: the proportion of participants who perceived pre-pregnancy overweight/obesity. The proportion of the classification is visually depicted by the size of the circle.

As shown in Figure 1, more than half of postpartum women (64.8%) perceived their PPW to be normal, 11.6% perceived it as underweight, and 23.6% perceived it as overweight or obese. However, the actual situation was that 64.3% of individuals had a normal weight, 16.8% were underweight, and 18.9% were overweight or obese before pregnancy.

The assessment of agreement between maternal actual and perceived PPW showed that 9.7% of the postpartum women underestimated their PPW, 66.9% correctly perceived it, and the remaining 23.3% overestimated it. The Kappa coefficient was 0.366 (*p* < 0.001), which was considered fair.

### 3.3. Agreements Between Maternal Actual and Perceived GWG

**Figure 2:**
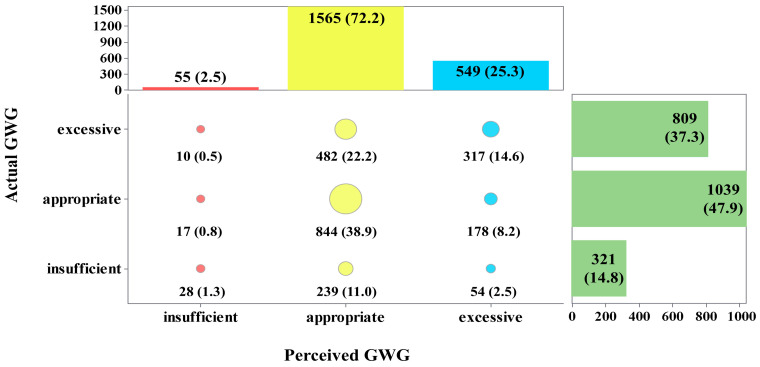
Proportions of exact agreement between actual and perceived GWG. GWG, gestational weight gain. Red circles: the proportion of participants who perceived insufficient GWG. Yellow circles: the proportion of participants who perceived appropriate GWG. Blue circles: the proportion of participants who perceived excessive GWG. The proportion of the classification is visually depicted by the size of the circle.

As shown in Figure 2, more than half of postpartum women (72.2%) perceived their GWG as appropriate, 2.5% perceived it as insufficient, and 25.3% perceived it as excessive. However, the actual situation was that less than half (47.9%) of participants had appropriate GWG, 14.8% had insufficient GWG, and 37.3% had excessive GWG.

The assessment of agreement between maternal actual and perceived GWG showed that 23.5% of the postpartum women underestimated their GWG, 54.8% correctly perceived it, and the remaining 21.7% overestimated it. The Kappa coefficient was 0.188 (*p* < 0.001), which was considered slight.

### 3.4. Preliminary Analysis of the Associations Between Perceived Bias in PPW or GWG and EPDS Scores

As shown in Table 2, the median EPDS score for postpartum women who perceived normal PPW was 5, significantly lower than those who perceived underweight (*p* = 0.001) and overweight/obese (*p* < 0.001). Similarly, the median EPDS score for postpartum women who perceived appropriate GWG was 5, significantly lower than those who perceived insufficient GWG (*p* = 0.038) and excessive GWG (*p* < 0.001). Postpartum women who underestimated their PPW had significantly lower EPDS scores compared to those who overestimated it (*p* = 0.017). Furthermore, participants who underestimated their GWG had notably lower EPDS scores than both those who overestimated (*p* = 0.002) and those who correctly estimated it (*p* < 0.001).

### 3.5. Adjusted Associations of Maternal Weight Perception with EPDS Scores

In this study, there was no multiplicative interaction between actual PPW and actual GWG on PPD status (Figure S1). As shown in Table 3, in the unadjusted model, postpartum women who perceived their PPW to be underweight or overweight/obese had an increased risk of developing PPD status compared to those with a perceived pre-pregnancy normal weight. After adjusting the model, this result still remained statistically significant (*p* < 0.05), with the EPDS scores increased by 0.70 and 0.86 points for those who perceived their PPW as underweight or overweight/obese, respectively.

As shown in Table 4, in the unadjusted model, participants’ insufficient actual GWG, perceived insufficient or excessive GWG, and underestimation or overestimation of GWG all had an impact on their risk of developing PPD status. However, in the adjusted model, only postpartum women who perceived their GWG as excessive had an EPDS score that was 0.47 points, significantly higher than that of those who perceived their GWG as appropriate (*β* = 0.47, *p* = 0.028).

**Figure 3:**
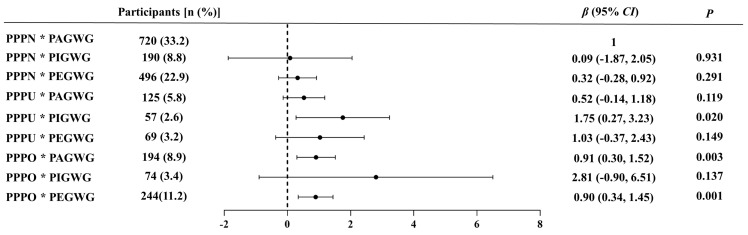
The multiplicative interaction between PPW perception and GWG perception on PPD status. * Indicates an interaction effect between variables; PPW, pre-pregnancy weight; GWG, gestational weight gain; PPD, postpartum depression; PPPN, perceived pre-pregnancy normal weight; PPPU, perceived pre-pregnancy underweight; PPPO, perceived pre-pregnancy overweight/obesity; PAGWG, perceived appropriate GWG; PIGWG, perceived insufficient GWG; PEGWG, perceived excessive GWG. Adjusted for maternal postpartum age, region of residence, household income, education level, physical activity level, and parity.

The multiplicative interaction results indicated that perceived abnormal PPW and inappropriate GWG had a positive multiplicative interaction on PPD status. After adjusting for covariates, participants who perceived their PPW as normal and their GWG as appropriate were considered as controls. Results showed that those who perceived their PPW as underweight and their GWG as insufficient (*β* = 1.75, *p* = 0.020), those who perceived their PPW as overweight and their GWG as appropriate (*β* = 0.91, *p* = 0.003), as well as those who perceived their PPW as overweight/obese and their GWG as excessive (*β* = 0.90, *p* = 0.001), had a significantly increased risk of developing PPD status (Figure 3).

**Figure 4:**
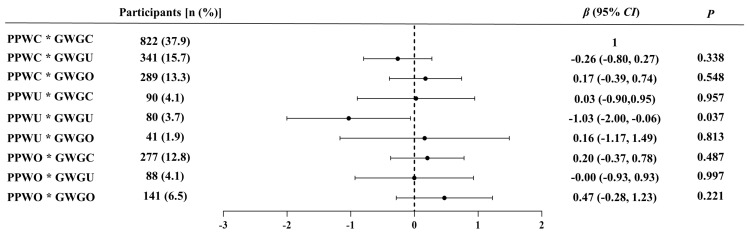
The multiplicative interaction between actual and perceived agreement of PPW and that of GWG on PPD status. * Indicates an interaction effect between variables; PPW, pre-pregnancy weight; GWG, gestational weight gain; PPD, postpartum depression; PPWC, PPW consistency; PPWU, PPW underestimation; PPWO, PPW overestimation; GWGC, GWG consistency; GWGU, GWG underestimation; GWGO, GWG overestimation. Adjusted for maternal postpartum age, region of residence, household income, education level, physical activity level, and parity.

The multiplicative interaction between actual and perceived agreement of PPW and that of GWG on PPD status is shown in Figure 4. After adjusting for covariates, participants who accurately perceived their PPW and GWG were considered as controls. Results only revealed a significant negative correlation between underestimating PPW and GWG with PPD status (*β* = −1.03, *p* = 0.037).

## 4. Discussion

This study innovatively explored the relationship between weight perception (including perceived weight and the agreement between actual and perceived weight) and PPD status, adjusting for other potential factors that could affect the risk of PPD status. The findings revealed that women who perceived their PPW or GWG as abnormal were more likely to experience PPD status. Meanwhile, the agreement between the actual and perceived values of PPW or GWG was poor. Specifically, women who underestimated both PPW and GWG were less likely to experience PPD status.

This study found that postpartum women under the age of 35, primiparity, living in urban areas, with a family per capita monthly income between RMB 5000 and RMB 10,000, a higher education level, and a low physical activity level had a higher risk of PPD status. In previous studies, the association between age and PPD was inconsistent. Bradshaw H’s study [26] found that depressive symptoms decreased with age, and the incidence rate of postpartum depressive symptoms among primiparous women was higher compared to multiparous women, which was consistent with our findings. However, some studies considered that older mothers were at a higher risk of PPD, with younger age being linked to a higher risk of PPD only among postpartum women without a prior history of depression [33]. Additionally, another study showed that there was no association between maternal age and EPDS scores [34]. Similar to our study, Mori E et al. found that during the first postpartum month, primiparous women of all ages experienced greater fatigue and higher risk of PPD status compared to multiparous women, with the oldest multiparous women being the most severely affected [35]. Primiparous women often face more anxiety and depression, potentially due to fear of childbirth, difficulties in breastfeeding, and lack of experience in neonatal care [36]. A study in Indonesia revealed a higher prevalence of PPD in urban areas compared to rural ones within the first 6 postpartum months [37], which was consistent with our findings. This may be due to the increased competitive pressures and unemployment risks faced by urban women after childbirth [38]. Most existing studies suggested that higher education levels and better living conditions were protective factors against PPD status [39,40], which was not fully consistent in this study. Zhao J et al. found that for women working in commercial enterprises, only a family per capita monthly income between RMB 5000 and RMB 15,000 was positively correlated with PPD [38], which was similar to the results of our study. Additionally, it is well known that moderate physical activity can reduce the risk of PPD status [41,42], which had been demonstrated in this study.

In this study, 18.9% of women were actually underweight before pregnancy, 64.3% were normal, and 16.8% were overweight or obese. In 2021, another study of 6223 women in Southern China showed that the proportion of women who were underweight before pregnancy was 19.3%, which was close to the proportion of women who were overweight or obese before pregnancy (19.4%) [43]. According to PPW, the optimal range for GWG was determined. In our study, 47.9% of participants had appropriate GWG, 14.8% experienced insufficient GWG, and 37.3% experienced excessive GWG. Data from the Ma’anshan Birth Cohort in China showed that the proportion of women with excessive GWG was higher than that in our study. The proportions of women with insufficient, appropriate, and excessive GWG were 8.2%, 33.3%, and 58.5%, respectively [44]. The differences may be due to variations in geographical location and economic conditions [45]. Studies showed that higher levels of PPW were predictive of decreased GWG and increased 6-month postpartum depressive symptoms [46]. Furthermore, both excessive and insufficient GWG were significantly associated with a higher risk of developing PPD status [47]. Previous studies confirmed that excessive GWG acted as a warning signal for symptoms of anhedonia and anxiety, whereas inadequate GWG was a crucial indicator of depressive symptoms [48]. However, after adjusting for covariates, this study did not find a statistically significant association between inappropriate GWG and PPD status.

In this study, 35.2% of women were dissatisfied with their PPW, with 11.6% considering themselves underweight and 23.6% perceiving themselves as overweight or obese before pregnancy. Additionally, 27.8% of women were dissatisfied with their GWG, with 2.5% feeling that their weight gain was insufficient and 25.3% believing it to be excessive weight during pregnancy. Statistical data indicated that women generally preferred a slimmer body image. Research by Rebecca A. et al. also demonstrated that 92% of women were dissatisfied with their pre-pregnancy body image, with 69% considering their PPW to be excessive [49]. A study from Japan indicated that women believed that avoiding excessive GWG could help prevent stretch marks, reduce postpartum weight retention, and alleviate labor pain, leading them to often pursue lower ideal GWG values [50]. The formation of these perceptions may be attributed to cultural and societal expectations regarding body image and health before and during pregnancy [51]. In addition, the findings of our study indicated that women’s subjective perceptions of inappropriate PPW and GWG significantly influence their risk of PPD status. Although there is no direct evidence linking weight perception to PPD, previous studies suggested that women dissatisfied with their pregnancy body image were at a higher risk of perinatal depression [52,53], which was consistent with our results. Conversely, positive body image perception is associated with higher self-esteem, more effective coping mechanisms, and overall psychological well-being, which can mitigate the risk of depressive symptoms in new mothers [54]. The results of this study further revealed that abnormal perceptions of PPW and GWG had interactive effects on the occurrence of PPD status. Specifically, women who perceived themselves as underweight before pregnancy and simultaneously perceived their GWG as insufficient faced notably higher risks of PPD status. Similarly, those who perceived themselves as overweight or obese before pregnancy, coupled with the perception of excessive GWG, were also significantly more likely to be at risk of PPD status. These findings represent a unique perspective, as previous studies have neglected to delve into this particular relationship.

Weight perception is an important determinant of dietary behavior and weight management practices. In our study, notable divergences were observed between women’s actual PPW or GWG and their perceptions of these weights. Specifically, the proportion of women who mistakenly perceived PPW and GWG was 33% and 45.2%, respectively. A study by Berenson AB et al. among 1420 women revealed that over 30% of those intending to become pregnant mistakenly perceived their weight [55]. In another study of 526 pregnant women in the United Arab Emirates, only 26.8% of them accurately perceived their GWG [56], which was much lower than our findings. In previous studies, there have been few similar studies on the impact of women’s misperceptions of PPW and GWG on PPD. Only one study of 717 nulliparous women of normal weight in Korea revealed that those who misperceived themselves as overweight/obese were more likely to engage in unhealthy weight management practices, display depressive symptoms, and experience psychological distress [57]. Individuals who were dissatisfied with their weight and attempted to correct it might suffer greater health risks than those who simply misperceive their weight [11]. In our study, the statistical significance of the association between weight misperception and PPD was observed in the univariate analysis; however, it was not present after adjusting for confounding variables. Our findings also suggested that underestimating both PPW and GWG might be a protective factor against PPD status. Contemporary society and culture exert a profound influence on individuals’ comprehension and perception of body image. Taking the portrayal of the ideal pregnancy body image by the media as an example, it often focuses on showcasing images of those with slightly bulging abdomen but still slender body images [58]. Women who underestimated their PPW and GWG may be less affected by such information, resulting in reduced anxiety about weight or body image recovery, increased body appreciation, and heightened self-identification. This, in turn, can contribute to better psychological health [59].

This study has several limitations. Firstly, due to its cross-sectional study design, it is challenging to directly establish a causal link between maternal weight perception and PPD status. Therefore, well-designed prospective studies will be necessary. Secondly, the scope of this study was limited to the maternal population in Southern China, which undoubtedly weakened the broad applicability of the study’s results to the maternal population nationwide. Finally, the assessment of PPD status in this study was solely based on self-reported data from the EPDS. Although this scale is widely recognized [60], it still lacks other objective diagnostic indicators or clinical benchmarks, which introduces the potential risk of information bias in the findings.

## 5. Conclusions

Women who perceived their weight before and during pregnancy as inappropriate were more prone to PPD status in our study. Therefore, it is crucial to help women develop a correct understanding of weight before and during pregnancy. While regular weight monitoring is beneficial for maternal and infant health, excessive focus on weight may trigger anxiety and depressive symptoms in some women. This suggests that health education on weight management for pregnant women should be personalized. For those predisposed to weight dissatisfaction, it is imperative to offer psychological support to cultivate a positive body image and self-esteem. In addition, specific and practical weight control-related strategies, such as nutritional meal recipes and pregnancy yoga classes, should be provided to assist women in achieving an ideal PPW and GWG. It is hoped that healthcare professionals and women will come together to develop women’s accurate perception of their weight. Future research can also objectively evaluate PPD through clinical diagnosis by professional doctors and psychological assessment tools. More extensive cohort studies are needed in the future to confirm the results of this study. It is also possible to comprehensively evaluate the impact of weight management before and during pregnancy on PPD, which can help to take more targeted and effective intervention measures.

## Figures and Tables

**Table 1:** Sociodemographic characteristics of postpartum women.

Characteristics	Total (*n* = 2169)	PPD (*n* = 391)	Non-PPD (*n* = 1778)	χ^2^	*p*
**Postpartum age (years)**				9.402	**0.002**
<35	1812 (83.5)	347 (88.7)	1465 (82.4)		
≥35	357 (16.5)	44 (11.3)	313 (17.6)		
**Ethnicity**				2.367	0.124
Han	1801 (83.0)	335 (85.7)	1466 (82.5)		
Minority	368 (17.0)	56 (14.3)	312 (17.5)		
**Region of residence**				37.157	**<0.001**
Urban	1051 (48.5)	244 (62.4)	807 (45.4)		
Rural	1118 (51.5)	147 (37.6)	971 (54.6)		
***Per capita* monthly income (RMB)**				33.742	**<0.001**
<5000	1167 (53.8)	165 (42.2)	1002 (56.4)		
5000~10,000	595 (27.4)	151 (38.6)	444 (25.0)		
>=10,000	407 (18.8)	75 (19.2)	332 (18.6)		
**Educational level**				6.522	**0.038**
Senior high school and below	1105 (50.9)	177 (45.3)	928 (52.2)		
Junior college/vocational university	551 (25.4)	107 (27.4)	444 (25.0)		
Bachelor’s degree or above	513 (23.7)	107 (27.4)	406 (22.8)		
**Employment**				1.171	0.279
Unemployed	952 (43.9)	162 (41.4)	790 (44.4)		
Employed	1217 (56.1)	229 (58.6)	988 (55.6)		
**Parity**				13.687	**<0.001**
Primiparous	1070 (49.3)	226 (57.8)	844 (47.5)		
Multiparous	1099 (50.7)	165 (42.2)	934 (52.5)		
**Mode of delivery**				1.124	0.289
Spontaneous labor	1540 (71.0)	269 (68.8)	1271 (71.5)		
Cesarean section	629 (29.0)	122 (31.2)	507 (28.5)		
**Gestational age at birth**				0.505	0.478
Preterm	260 (12.0)	51 (13.0)	209 (11.8)		
Term	1909 (88.0)	340 (87.0)	1569 (88.2)		
**Passive smoking status**				0.073	0.788
Yes	154 (7.1)	29 (7.4)	125 (7.0)		
No	2015 (92.9)	362 (92.6)	1653 (93.0)		
**Physical activity levels**				44.091	**<0.001**
High/moderate	1161 (53.5)	150 (38.4)	1011 (56.9)		
Low	1008 (46.5)	241 (61.6)	767 (43.1)		

Data are expressed as *n* (%). PPD, postpartum depression. Bold values indicate a statistically significant difference in results.

**Table 2:** Preliminary analysis of the associations between perceived bias in PPW or GWG and EPDS scores.

Weight Perception	*n*	EPDS Scores	Z	*p*
**Perceived PPW**			43.350	**<0.001**
Underweight	251	6 (4, 10)		
Normal weight	1406	5 (3, 9)		
Overweight/obesity	512	7 (4, 10)		
**Perceived GWG**			21.080	**<0.001**
Insufficient	55	7 (4, 10)		
Appropriate	1565	5 (3, 9)		
Excessive	549	6 (4, 10)		
**Agreement of actual and perceived PPW**			6.332	**0.042**
Underestimation	211	5 (3, 9)		
Consistency	1452	6 (3, 9)		
Overestimation	506	6 (3, 9)		
**Agreement of actual and perceived GWG**			17.130	**<0.001**
Underestimation	509	5 (3, 8)		
Consistency	1189	6 (3, 9)		
Overestimation	471	6 (3, 10)		

Data are presented as median (*P*_25_, *P*_75_). PPW, pre-pregnancy weight; GWG, gestational weight gain; EPDS, Edinburgh Postnatal Depression Scale. Bold values indicate a statistically significant difference in results.

**Table 3:** Adjusted associations of actual PPW, perceived PPW, and the results of their agreement with EPDS scores.

Characteristics	Unadjusted Model		Adjusted Model	
	*β* (95% *CI*)	*p*	*β* (95% *CI*)	*p*
**Actual PPW**				
Normal weight	Ref.		Ref.	
Underweight	0.15 (−0.35, 0.64)	0.566	0.19 (−0.27, 0.66)	0.441
Overweight/obesity	0.19 (−0.33, 0.70)	0.484	0.17 (−0.32, 0.66)	0.502
**Perceived PPW**				
Normal weight	Ref.		Ref.	
Underweight	0.97 (0.37, 1.57)	**0.001**	0.70 (0.13, 1.27)	**0.016**
Overweight/obesity	1.32 (0.87, 1.77)	**<0.001**	0.86 (0.43, 1.29)	**<0.001**
**Agreement of actual and perceived PPW**				
Consistency	Ref.		Ref.	
Underestimation	−0.50 (−1.15, 0.14)	0.127	−0.32 (−0.94, 0.29)	0.302
Overestimation	0.39 (−0.06, 0.85)	0.090	0.27 (−0.16, 0.70)	0.219

Adjusted model: adjusted for maternal postpartum age, region of residence, household income, education level, physical activity level, and parity. PPW, pre-pregnancy weight; EPDS, Edinburgh Postnatal Depression Scale; CI, confidence interval. Bold values indicate a statistically significant difference in results.

**Table 4:** Adjusted associations of actual GWG, perceived GWG, and the results of their agreement with EPDS scores.

Characteristics	Unadjusted Model		Adjusted Model	
	*β* (95% *CI*)	*p*	*β* (95% *CI*)	*p*
**Actual GWG**				
Appropriate	Ref.		Ref.	
Insufficient	0.91 (0.35, 1.47)	**0.001**	0.39 (−0.14, 0.92)	0.151
Excessive	0.12 (−0.29, 0.54)	0.553	−0.02 (−0.41, 0.37)	0.937
**Perceived GWG**				
Appropriate	Ref.		Ref.	
Insufficient	1.24 (0.03, 2.44)	**0.044**	1.11 (−0.02, 2.25)	0.054
Excessive	0.92 (0.48, 1.35)	**<0.001**	0.47 (0.05, 0.88)	**0.028**
**Agreement of actual and perceived GWG**				
Consistency	Ref.		Ref.	
Underestimation	−0.61 (−1.08, −0.15)	**0.010**	−0.39 (−0.83, 0.05)	0.085
Overestimation	0.50 (0.03, 0.98)	**0.039**	0.21 (−0.24, 0.67)	0.356

Adjusted model: adjusted for maternal postpartum age, region of residence, household income, education level, physical activity level, and parity. GWG, gestational weight gain; EPDS, Edinburgh Postnatal Depression Scale; CI: confidence interval. Bold values indicate a statistically significant difference in results.

## Data Availability

The data utilized in this study are available upon request from the corresponding author.

## Supplementary Materials

The following supporting information can be downloaded at https://www.mdpi.com/article/10.3390/nu16213696/s1, Figure S1: The multiplicative interaction between actual PPW and actual GWG on PPD status.
