# Supplementary material for: Association Between Maternal Weight Perception Before and During Pregnancy and Postpartum Depression Status in Southern China
Source: Nutrients. 2024 Oct 30;16(21):3696. doi: 10.3390/nu16213696 (PMC11547851; doi:10.3390/nu16213696)
Supplement: Supplementary file 1 [file nutrients-16-03696-s001.zip › nutrients-3284305-Supplementary.pdf]

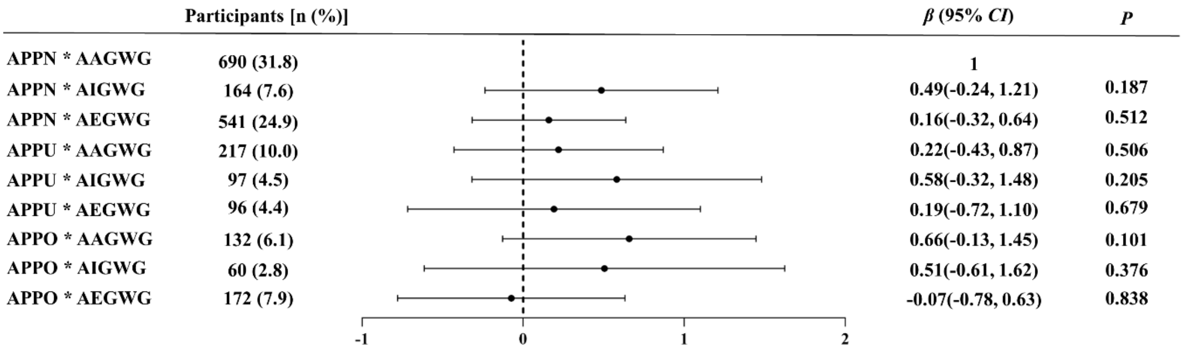

Figure S1: The multiplicative interaction between actual PPW and actual GWG on PPD status

*Indicates an interaction effect between variables; PPW, pre-pregnancy weight; GWG, gestational weight gain; PPD, postpartum depression; APPN, actual pre–pregnancy normal weight; APPU, actual pre–pregnancy underweight; APPO, actual pre–pregnancy overweight/obesity; AAGWG, actual appropriate GWG; AIGWG, actual insufficient GWG; AEGWG, actual excessive GWG. Adjusted for maternal postpartum age, region of residence, household income, education level, physical activity level and parity.
